# Recontextualizing the subject of phenomenological psychopathology: Establishing a new paradigm case

**DOI:** 10.3389/fpsyt.2022.1035967

**Published:** 2022-10-21

**Authors:** Guilherme Messas, Anthony Vincent Fernandez

**Affiliations:** ^1^Santa Casa de São Paulo School of Medical Sciences, São Paulo, Brazil; ^2^Collaborating Centre for Values-Based Practice in Health and Social Care, St. Catherine's College, University of Oxford, Oxford, United Kingdom; ^3^Department of Sports Science and Clinical Biomechanics, University of Southern Denmark, Odense, Denmark; ^4^Danish Institute for Advanced Study, University of Southern Denmark, Odense, Denmark

**Keywords:** phenomenological psychopathology, phenomenological psychiatry, substance use disorder, phenomenology, situatedness, context

## Abstract

Recently, there have been calls to develop a more contextual approach to phenomenological psychopathology—an approach that attends to the socio-cultural as well as personal and biographical factors that shape experiences of mental illness. In this Perspective article, we argue that to develop this contextual approach, phenomenological psychopathology should adopt a new paradigm case. For decades, schizophrenia has served as the paradigmatic example of a condition that can be better understood through phenomenological investigation. And recent calls for a contextual approach continue to use schizophrenia as their primary example. We argue, in contrast, that substance misuse provides a better paradigm case around which to develop a contextually sensitive phenomenological psychopathology. After providing a brief vignette and analysis of a case of substance misuse, we explain why this kind of condition requires considerable sensitivity and attention to context, better motivating the incorporation and development of new contextually sensitive approaches.

## Introduction

Phenomenologists, like most philosophers, are often accused of being too abstract. When articulating fundamental structures of subjectivity and the lived world, they often lose sight of how experience can also be shaped by one's socio-cultural context. For a philosopher concerned with experience in general, the acontextual nature of their investigation may be a feature—not a flaw—of their approach. However, the accusation of abstraction becomes more compelling when we turn to fields of applied phenomenology, which often aim to understand the experiences of particular subjects, rather than experience in general.

This is certainly the case for the field of phenomenological psychopathology, which aims to understand and describe experiential alterations that occur in mental illness. When describing the experiences of people living with schizophrenia, for instance, one makes claims about a particular population, rather than about experience in general. However, contemporary phenomenological psychopathologists often develop their accounts without much sensitivity to the socio-cultural context of the people they study. In most cases, phenomenological psychopathologists take themselves to be investigating alterations in fundamental structures of experience. Research on schizophrenia, for instance, has focused on the disturbances of minimal selfhood, or the basic sense of “for-me-ness” that is said to accompany all experience (1, 2). And research on melancholic depression has focused on disturbances of implicit temporality, such as diminishment of the conative drive, or the implicit urge or striving toward the future (3). There seems to be a general assumption that these structural alterations are not shaped by cultural, linguistic, or religious background, life circumstances, and so on. This still leaves room for various contextual factors to fill in the *content* of experience—for instance, the content of delusions or hallucinations among people with schizophrenia can vary from culture to culture. But the primary focus of phenomenological inquiry has typically been on more fundamental structural alterations, which are presumed to be untouched by cultural differences.

However, we do find some contextually sensitive approaches in both classical and contemporary phenomenological psychopathology [e.g., (4, 5)]. Recently, Elizabeth Pienkos outlined some of these approaches and argued that the field of phenomenological psychopathology as a whole should incorporate more sensitivity to context. She shows how classical phenomenological psychopathologists attended both to the particularities of individual patients, such as their psychological style or life history, as well as aspects of their lived world, such as social stressors, cultural values, and relations with others (6). Some contemporary approaches also incorporate more sensitivity to situation and context by drawing on insights from other approaches in philosophy and the sciences, such as the bio-psycho-social model (7), hermeneutics (8), or enactivism (9, 10).

We fully endorse a contextual approach as the right direction for the field. There is, however, another issue that we want to address here: For decades, phenomenological psychopathology has centered on the “paradigm case” of schizophrenia. Many of the general approaches and theoretical frameworks that now characterize the field as a whole were initially developed with the more specific aim of understanding this condition. Even Pienkos centers her proposal around the study of schizophrenia, showing how a contextual approach can help us understand it as a disorder of both self *and* world. We agree that these phenomenological accounts will be enhanced by a more contextual approach. However, we suggest that continuing to use schizophrenia as a paradigm case of mental illness may not be the most productive route to developing such an approach. Phenomenological work on schizophrenia may have been able to proceed in an acontextual manner because many of the core features of this condition *can* be understood without much attention to context. It may very well be the case that a contextual approach will provide a more nuanced understanding of schizophrenia. But the understanding that we've been able to attain with phenomenological psychopathology's current, more or less acontextual approach is already impressive.

If we truly want to develop a contextual approach to phenomenological psychopathology, why not start from another paradigm case—a condition that can be understood only with a high degree of sensitivity to both social and personal context? We argue that addiction, and substance misuse more broadly, may provide just such a paradigm case, precisely because it cannot be understood acontextually. To illustrate this, we provide a brief vignette and phenomenological analysis. The vignette is a fictionalized composite of substance misuse patients, intended to represent a typical case that should resonate with clinicians. Through this case, we show how the very idea of understanding substance misuse in an acontextual way is a non-starter. Only by attending to specific contextual details of the case—one's culture, upbringing, relations with friends, family, colleagues, environment, and so on—can the phenomenological psychopathologist begin to understand the relevant experiential alterations. To simplify the analysis, we focus exclusively on alterations in selfhood and intersubjectivity. In this respect, we highlight the self-other relation. However, a genuinely contextual approach will of course need to attend to the self-other-*world* relation (11). This would require that that the case be analyzed along other dimensions as well, attending more explicitly to temporal (12, 13) and spatial (14) alterations.

## Substance misuse illustrated through a vignette and analysis

Peter, 36 years old and married since he was 23, comes from a poor family marked by many cases of substance abuse, which led to a significant family breakdown. Due to his father's alcoholism, his mother, who was mainly responsible for his upbringing, moved several times between different places and cities, always looking for some stability for the upbringing of her children. Despite his difficult personal history, Peter never saw himself as a sad person. On the contrary, he describes himself as someone who always liked the streets, where he could release his often uncontrolled energy: “I always had a compulsion inside me, something I had to do, to unload. And I didn't know what I had to do, how to control it. I didn't have orientation, someone to say, look, go to a psychologist, so he can orient you on how to spend your energy there.” At the age of 16, he started using cannabis, initially aiming to reduce his agitation. He says the fact that his cousins were also using substances made this choice easier. Gradually, he started using other drugs, until he found crack cocaine, with which he lost control and started using it compulsively. He says that crack cocaine is “...very strong, it causes that thing, that ecstasy in you, and the next day you have the need to smoke it again.” He always interpreted his misuse as contrary to his personal values: “Even though this feeling [of intoxication] was so strong inside me (the interviewee cries) the next day I would try to use again. So I saw that it was stronger than me. I was losing.”He underwent many treatments over the years, alternating periods of control with new and harmful relapses.

Peter is very clear about the intersubjective context related to the loss of control, stating,

“(...) the only thing I'm afraid of, if I'm going to talk like this, is having a person in my life who doesn't have emotional control. Because I, since I have a problem with emotions, that I think what releases our compulsion, of people like me, that this predisposition is the oscillations of the sentimental, of emotions, so I have to be with a person who is emotionally strong.”

In his specific case, he affirms, “My recovery is very much linked to the fellowship that I follow, of narcotics anonymous, which is me, God, and the society that I serve.” He also describes precisely what he considers to be recovery from addiction: “The person begins to feel his values again. The emotions come out, the values, family, work, the intellectual part, it all comes back to him. The limits he must have, what he must do, so all this puts the man back in the center. I started to miss my wife and daughter again, to value everything I had.”

The above vignette immediately highlights Peter's relationships with the people close to him. While the nature of these relationships may be difficult to describe, many would characterize them as unhealthy or out of balance. From a phenomenological perspective, we can say that they express disproportions in intersubjectivity (15). This is not to suggest that the general or fundamental structure of intersubjectivity is altered (e.g., through a diminished capacity to empathically perceive others). Rather, the relevant disproportion or disturbance operates at a less fundamental level—which is not to suggest that its effects on one's life are any less significant (16).

Peter seems to rely heavily on others when making decisions. Even the simplest decision about his own life constitutes something difficult and risky. He experiences the decision-making process as something too complex to wade through alone. As a result, his autonomy has been gradually replaced by a kind of heteronomy, whereby it becomes difficult for him to control or determine the pathways of his own biographical development or life narrative. Giving someone else authority over the direction of his own life, over his daily decision-making processes, provides a degree of stability and comfort. But granting this kind of authority does, of course, come with its own dangers. His lack of autonomy not only comes with a distressing sense of emptiness, but also makes him exceptionally vulnerable to abuse. Without a strong sense of autonomy and sense of self, he relies on others to help make his day-to-day decisions, but also relies on their view of him to constitute his own value and identity. Ideally, our experiential poles of self and other should be in proportion, allowing the views of others to have some effect on our sense of self and identity while also retaining a degree of autonomy over who we take ourselves to be and what we choose to do with our lives.

One should not, however, understand this disproportion only in a negative perspective, as deprivation of the self. As illustrated above, the right person can help to guard and preserve one's own values; their direction is, effectively, a guiding action that respects and preserves one's own values. That is why mutual help groups are fundamental in the recovery of people with addiction or problems with substance misuse, yielding therapeutic outcomes (17, 18). Through this form of intersubjective association, the emptied person can find someone who, having experienced the same condition, is able to temporarily take the place of a strong person to lead their life. The emptied self may recover through the influence of a strong other who helps them establish and maintain minimally stable values.

But we also see another aspect of intersubjective disproportion in this case: a profound existential submission to a collective, whereby the self can be determined entirely by others. At times, Peter is imprisoned by how the collective values (or fails to value) him. In this disproportion, the self is too sensitive to the perceptions of the collective. Acting in a way that doesn't accord with the collective's values brings about intense guilt. There is thus a direct link between the frequent experiences of guilt of a person with substance misuse and their submission to the social, even in those who do not feel depressed and even take pleasure from the experience of marginality (19). When a person's existence is characterized by this kind of submission, they are pre-reflexively conditioned by set social duties whose values they cannot relativize. They are too exposed to the values prescribed to them and are unable to establish enough distance to constructively reflect on and revise them.

## Discussion

How does the analysis of this vignette differ from traditional phenomenological analyses of other conditions, such as schizophrenia? It's not simply that the vignette provides extensive biographical details. In principle, an analysis of schizophrenia could also start from a biographically rich vignette. Rather, the major difference is that an accurate analysis of experiences of addiction and substance misuse requires a constant sensitivity to this kind of biographical context. This kind of approach allows us to consider how and why the person's behavior and experiences differ so dramatically from one context to another. We see, for instance, that when Peter has someone who helps him establish and maintain a strong value system, he can live a life free of substance misuse. In the right context, we may not even perceive him as ill. Without these interpersonal relationships, he falls back into the same harmful behavior. But this is not entirely the product of context, since many people do not fall into these behaviors when placed in the same contexts. Rather, a genuinely contextual approach to phenomenological psychopathology is one that takes seriously how our self-other-world relations shape the very form or structure of our experience.

In this example, the specific kinds of relationships that Peter developed (or failed to develop) with individuals and collectives, as well as his own reflections on the purpose and role of these relationships, are what provides genuine insight into how his experiential life is structured. We have argued that he exhibits an anthropological disproportion in intersubjectivity, where the pole of the other is weighted much more heavily than the pole of the self. And this is precisely why his interpersonal circumstances have the effects on him that they do.

This kind of disproportion, or experiential alteration, also highlights another reason that addiction and substance misuse require a contextually sensitive analysis. Phenomenological studies of psychopathological conditions are often concerned with deep or fundamental alterations in the structure of experience. We might characterize some of these alterations as occurring at the ontological level—that is, they involve changes in what the classical phenomenologists considered to be essential, even invariant, structures of experience and subjectivity (20, 21). However, what we have referred to here as the anthropological level (and might also be called the ontic or empirical level), includes aspects of experience and subjectivity that are less fundamental and more variable from person to person. While these differences and alterations are not as fundamental as, for instance, the loss of conative drive in melancholic depression or the disturbance of minimal selfhood in schizophrenia, they're key to understanding a variety of conditions.

These anthropological disproportions play a central role in the study of substance misuse, as they can inform our understanding of the specific vulnerabilities that lead some people to misuse substances. Identifying these disproportions helps to clarify the meaning that each experience of intoxication has within a person's lived world and understand why intoxication is so attractive to them (22). We can briefly illustrate this point with a particular personality type that is often vulnerable to substance misuse: borderline personality (23). For people with this personality type, the poles between self and other are also weighted heavily toward otherness. Others are therefore able to influence the borderline self in a way that can make it unstable. For example, a person with borderline personality may find it difficult to maintain the same dispositions or life plans when a person who played a dominant role in their life is no longer directly present. To this kind of personality, misusing substances may serve as a viable coping strategy because these substances can provide affective continuity, which allows one to sustain the affective disposition they need to continue one's life projects. Intoxication can therefore compensate for the instability that typically arises out of one's own disproportions.

By taking a contextual approach and attending to conditions that are primarily characterized by disproportions or alterations at the anthropological level, we also arrive at insights that may have more immediate clinical relevance. For instance, a rich understanding of how the structure of a patient's experience shapes his relations and interactions with others is an invaluable resource, not only for the traditional psychotherapeutic encounter, but also for clinical decision-making processes about the most effective programs for treatment and care (14, 24). The vignette and analysis, for example, highlights precisely why narcotics anonymous was an effective therapeutic intervention for him. To understand its effectiveness, we need to attend not only to narcotics anonymous as a specific context that he's placed in (perhaps at the suggestion of his psychiatrist), but also to Peter's personal sensitivities to context—to how he relates to collectives by adopting and incorporating their values.

This is also where a greater focus on Peter's material environment would help us understand his experience and behavior. The social environment of narcotics anonymous may, for instance, be in tension with Peter's material environment, which might provide him with easy access to narcotics. A complete analysis of Peter's case would therefore require a more holistic account of self-other-world relations. While classical phenomenology certainly provides resources for thinking about the role of one's material environment, this may also be an opportunity to incorporate more ecological or enactive approaches to analyze the nature and role of material environments, including how environments affords us a space of possibilities.

If we truly want to pursue a more contextual approach to phenomenological psychopathology, where do we go from here? Adopting a new paradigm case that refocuses our attention on the role of context is only a means toward a further end. It highlights the need for new tools and approaches that help phenomenology better contribute to current needs in mental healthcare. While it's true that some phenomenological psychopathologists have used contextual approaches, many other phenomenological traditions have developed considerably more robust ways of attending to context and socio-cultural situatedness. Within philosophy, we might draw from fields such as feminist phenomenology, phenomenology of race, phenomenology of disability, and critical phenomenology [e.g. (25)]. And, if we look beyond philosophy, to phenomenological approaches developed across the human and social sciences, we also find more empirical applications of phenomenology, such as integrations with qualitative and ethnographic methods [e.g., (26–30)]. We believe that the time is ripe for more integrative approaches to phenomenology, drawing on the rich methodological tools developed across phenomenology's diverse traditions. And closer attention to cases of addiction and substance misuse can facilitate the integration of these approaches with phenomenological psychopathology because they require careful attention to various dimensions of context.

## Data availability statement

The datasets presented in this article are not readily available because clinical interview data is not available to be shared publicly. Requests to access the datasets should be directed to guilherme.messas@fcmsantacasasp.edu.br.

## Author contributions

All authors listed have made a substantial, direct, and intellectual contribution to the work and approved it for publication.

## Funding

Open access funds provided by the Danish Institute for Advanced Study, University of Southern Denmark.

## Conflict of interest

The authors declare that the research was conducted in the absence of any commercial or financial relationships that could be construed as a potential conflict of interest.

## Publisher's note

All claims expressed in this article are solely those of the authors and do not necessarily represent those of their affiliated organizations, or those of the publisher, the editors and the reviewers. Any product that may be evaluated in this article, or claim that may be made by its manufacturer, is not guaranteed or endorsed by the publisher.
